# Probing into the Mechanism of Alkaline Citrus Extract Promoted Apoptosis in Pulmonary Fibroblasts of Bleomycin-Induced Pulmonary Fibrosis Mice

**DOI:** 10.1155/2018/9658950

**Published:** 2018-03-26

**Authors:** Qi Wu, Yao Zhou, Fan-chao Feng, Yi-han Jin, Zhi-chao Wang, Xian-mei Zhou

**Affiliations:** ^1^Affiliated Hospital of Nanjing University of Chinese Medicine, Nanjing 210029, China; ^2^Department of Respiratory Medicine, Jiangsu Province Hospital of Traditional Chinese Medicine, Nanjing 210029, China

## Abstract

We extracted the primary pulmonary fibroblasts of the normal and bleomycin-induced pulmonary fibrosis mice and investigated the functioning mechanism of citrus alkaline extract (CAE) in the induction of pulmonary fibroblast apoptosis. The expression intensity of vimentin of the pulmonary fibroblasts in the model mice was higher than that in the normal mice. Meanwhile, the positive expression rate and expression intensity of alpha smooth muscle actin (*α*-SMA) of the pulmonary fibroblasts in the model mice were higher than those in the normal mice. Results of MTT showed that pulmonary fibroblast activity of the normal and model mice has been significantly inhibited by CAE in a concentration-dependent manner. The results of flow cytometer analysis showed that the proportion of pulmonary fibroblast apoptosis in the model mice has been profoundly increased by CAE treatment in a dosage-dependent manner. Besides we found that the expression of Cleaved-Caspase 3, Cleaved-Caspase 8, Cleaved-poly-ADP-ribose polymerase (Cleaved-PARP), and Fas and Fas Ligand (FasL) was markedly increased after CAE treatment. A further study showed that the expression of Cyclooxygenase-2 (COX-2) and prostaglandin E receptor 2 (EP2) was dependant on the concentration of CAE, indicating that CAE-regulated receptor apoptosis of Fas was probably related to COX-2. The results of fluorescence detection of oxidative stress showed that the level of oxidative stress was significantly increased after CAE treatment. Furthermore, the results of Western Blot showed that the phosphorylation level of p38 (p-p38) was markedly increased, suggesting that CAE probably has regulated COX-2 through increased p-p38 following oxidative stress. Our results therefore suggest that CAE can effectively induce pulmonary fibroblast apoptosis of the normal and model mice, and its functioning mechanism is probably related to the p38/COX-2/Fas signaling pathway regulated by oxidative stress.

## 1. Introduction

Pulmonary fibrosis is a chronic diffusive interstitial lung disease [1]. The primary pathological feature is extracellular matrix deposition (ECM) that may cause abnormal remolding of pulmonary tissues, respiratory failure, and even death eventually [2]. It is now universally acknowledged that pulmonary fibroblast is the core step of pulmonary fibrosis and that effective intervention of the biological behavior of pulmonary fibroblasts can prevent and cure pulmonary fibrosis. Pulmonary fibroblasts proliferate and differentiate into myofibroblasts when pulmonary tissues were damaged, express specially alpha smooth muscle actin (*α*-SMA), overproduce ECM, and speed up the process of pulmonary fibrosis [3]. Therefore, removal of activated pulmonary fibroblasts and reduction of the secretion and deposition of ECM are effective tactics for the treatment of pulmonary fibrosis.

Currently, it is believed that accelerated pulmonary fibroblast apoptosis is a critical step for controlling the occurrence and development of pulmonary fibroblasts [4]. Domestically and internationally, a majority of researches concerning the control of pulmonary fibroblast activity concentrated on the inhibition of pulmonary fibroblast proliferation and collagen secretion. Although evolution of pulmonary fibrosis can be alleviated to a certain degree by this method, it is very difficult to completely inhibit its proliferation because of its high productivity [5]. Inhibition of collagen synthesis does not reduce the quantity of pulmonary fibroblasts and therefore cannot radically clear off pulmonary fibroblasts. The pulmonary fibroblast itself not only has strong antiapoptosis capacity, but also can induce the apoptosis of pulmonary epithelial cells and accelerate the evolution of pulmonary fibrosis [6]. Therefore, effective acceleration of pulmonary fibroblast apoptosis is of very important significance for the treatment of pulmonary fibrosis.

It has been confirmed that Fas-induced apoptosis signaling pathway plays a crucial role in pulmonary fibrosis [7]. Fas binds to FasL to form a death-induced compound. This compound activates Caspase 8 that induces Caspase 3 production and activates death receptor apoptosis pathway [8]. At present, it is believed that COX-2 plays an important role in regulating the expression of Fas receptor in pulmonary fibroblasts [9]. And COX-2 expression is related to the upstream oxidative stress-medicated MAPK signaling pathway [10]. We therefore speculate that Fas-induced pulmonary fibroblast apoptosis probably is closely related to the signaling pathway involved in MAPK/COX-2.

At present, there is no effective drug for pulmonary fibrosis. In early time, pulmonary fibrosis was treated by glucocorticoid, cyclosporin, colchicine, and cyclophosphamide. But they were all denied due to poor efficiency and big side effects. The validity and safety of long-term administration of the new drugs pirfenidone and nintedanib are ambiguous [11]. Therefore, we tried to seek for suitable medicine for pulmonary fibrosis from traditional Chinese medicine.

Citrus is the dry pericarp of ripe fruits of oranges and other cultivars of rutaceous plants. It is a frequently used traditional Chinese medicine with wide distribution, abundant resources, and high medical value. It has been confirmed that citrus and its active ingredient have functions in anti-inflammation and antioxidation, and they have been widely used in the prevention and treatment of cardiovascular and cerebrovascular diseases [12]. It was also reported that citrus had a certain effect in treating the ovalbumin-induced asthma model [13]. We previously studied the active ingredients against pulmonary fibrosis in citrus, and found that both the ethanol extract and alkaline extract had activities against pulmonary fibrosis* in vivo *and* in vitro* [14, 15]. However, the functioning mechanisms remain to be elaborated. Therefore, in this study, we extracted the primary pulmonary fibroblasts of pulmonary fibrosis mice, investigated the relation between CAE and pulmonary fibroblast apoptosis, and defined the functioning mechanism of CAE against pulmonary fibrosis by promoting pulmonary fibroblast apoptosis.

## 2. Materials and Methods

### 2.1. Reagents and Antibodies

Citrus was purchased from Jiangsu Hospital of Traditional Chinese Medicine (Nanjing, China). CAE was prepared according to a previous paper. The information of other reagents was as follows: bleomycin: Japan Chemical Plant; prednisone: Tianjin Pharmaceutical Co. Ltd., China; pirfenidone: Beijing Continent Pharmaceutical Co. Ltd., China; DMSO, fetal bovine serum, DMEM, and pancreatin: Sigma-Aldrich, USA; penicillin-streptomycin and LDH test kit: Nanjing Jiancheng Bioengineering Institute, China; Annexin V-FITC test kit: Becton, Dickinson and Company, USA; oxidative stress test kit: ENZO Biochem, USA; and antibodies against vimentin, *α*-SMA, Cleaved-Caspase 3, Cleaved-Caspase 8, Cleaved-PARP, p-p38, and *β*-tubulin: Cell Signaling Technology, USA. Antibodies against EP2, COX-2, Fas and FasL were from Abcam, England.

### 2.2. Pulmonary Fibrosis Model Construction

Male 6–8-week old C57BL/6 mice (SPF grade) were used in the experiment and their body weight was 18–20 g. The mice were supplied by Cavens Laboratory Animal Co., Ltd (Changzhou, China). These mice were reared in Traditional Chinese Medicine Research Institute for Pediatrics, Nanjing University of Traditional Chinese Medicine. These animals were acclimatized at room temperature for one week and supplied food and water ad libitum.

Mice were paralyzed by chloral hydrate through intraperitoneal injection; then pulmonary fibrosis mouse models were constructed by intratracheal instillation of 5 mg/kg bleomycin. Details about this method can be found in previous publication [15].

### 2.3. Cell Culture and Cell Viability Test

Primary pulmonary fibroblasts were extracted according to the method of Neveu et al. described [16]. Pulmonary fibroblasts were cultured in DMEM culture media containing 10% FBS and 1% penicillin-streptomycin. Cells in their exponential growth phase were tested after being digested by 0.25% pancreatin. The density of pulmonary fibroblasts was adjusted to 1 × 10^5^/ml. 100 *μ*l cell solution was added to a 96-well plate and then equal amount of CAE solution was supplemented. 48 hr later, pulmonary fibroblast activity was tested by MTT method. CAE was dissolved by DMSO, and the concentration of DMSO in each solution is ≤0.1%.

### 2.4. Identification of Pulmonary Fibroblast

Cells in their exponential growth phase were used to prepare cell-climbing slice. Immunohistochemical method was used to test the expression of vimentin and *α*-SMA.

### 2.5. LDH Release Test

We used LDH test kit to evaluate the toxicity of LDH. After testing by the foregoing method, the supernatant was collected and then tested according to the user's manual of the LDH test kit.

### 2.6. Apoptosis Test

After 24 hr of incubation, the pulmonary fibroblasts were inoculated to 6-well plates with a density of 1 × 10^5^/ml. These pulmonary fibroblasts were treated by low, middle, and high concentrations (50 ug/ml, 250 ug/ml, and 500 ug/ml) of CAE. 48 hr later, the cells were harvested and stained by Annexin V-FITC test kit. Cell apoptosis was tested with the flow cytometer and apoptosis was analyzed by the FlowJo software. In addition, cell-climbing slices were prepared under the foregoing condition. The slices were stained by Annexin V-FITC test kit, observed with a fluorescence microscope, and analyzed with by image J software.

### 2.7. Intracellular Oxidative Stress Level Test

Cells were treated by the foregoing condition. The original culture media were discarded and cells were rinsed three times with PBS. The oxidative stress level was tested according to user's manual of the test kit.

### 2.8. Western Blot Analysis

Pulmonary fibroblasts were lysed by the proteinase inhibitor, RIPA buffer. Protein concentration was determined by BCA test kit, boiled 10 min in SDS, and then saved at −80°C. The protein was separated by 8%~12% SDS PAGE and then transferred to PVDF film. The nonspecific antigen was sealed by 5% BSA for 1.5 h at room temperature. Diluted corresponding antibody (1 : 1000) was added and then left at 4°C overnight. The film was washed three times with 1% TBST, and each washing was 10 min. The secondary antibody (1 : 2000) was then added and the resulting mixture was incubated one hour at room temperature. The film was then washed three times with 1% TBST again and each washing was 10 min. Color developing reagent was added and then salaried in dark.

### 2.9. Statistical Analysis

All data were processed by SPSS18.0 software package. The data were expressed as $\overset{-}{X}\pm S$. Between-group difference was tested by the univariate analysis of variance. *P* < 0.05 was considered statistically significant.

## 3. Results

### 3.1. Identification of Pulmonary Fibroblast

**Figure 1:**
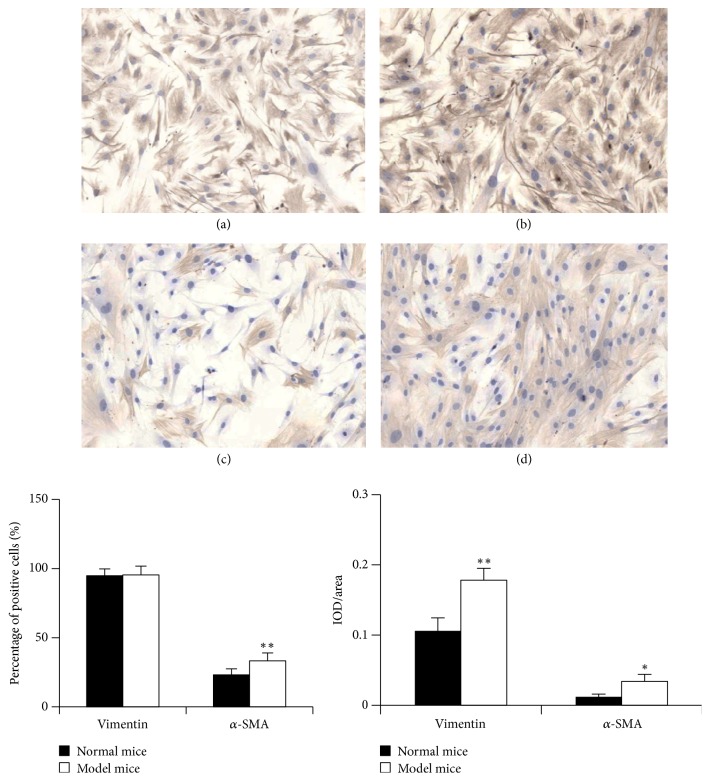
Identification of pulmonary fibroblast: cells in their exponential growth phase were harvested and used to prepare cell-climbing slice. Examples of immunohistochemical staining patterns for vimentin and *α*-SMA in the primary pulmonary fibroblasts at a total magnification of 100x. (a) Vimentin in the normal mice; (b) vimentin in the model mice; (c) *α*-SMA in the normal mice; (d) *α*-SMA in the model mice. For the statistics of each panel in this figure, all data are expressed as mean ± SD (*n* = 5). ^*∗*^*P* < 0.05 versus normal mice, ^*∗∗*^*P* < 0.01 versus normal mice.

Under normal circumstances, vimentin was only expressed in mesenchymal tissue but not expressed in epidermal cells [17]. Therefore vimentin can be the maker protein of fibroblasts. *α*-SMA is considered as the marker of myofibroblast and interstitial cell. We extracted the primary pulmonary fibroblasts of the normal and model mice, respectively, and then analyzed comparatively both fibroblasts through immunohistochemical staining of vimentin and *α*-SMA. As shown in Figure 1, in both fibroblasts the cytoplasm was stained into light brown when vimentin was subject to immunohistochemical staining, which suggests a positive expression and successful cell extraction. Although the positive expression rate of vimentin in both fibroblasts did not differ significantly, the expression intensity of pulmonary fibroblasts in the model mice was higher than that in the normal mice. The positive expression rate and expression intensity of *α*-SMA of the pulmonary fibroblasts in the model mice were also higher than those in the normal mice. Furthermore, the bleomycin-induced mouse pulmonary fibroblast was much closer to human pulmonary fibroblast, which facilitates studies.

### 3.2. Inhibition of Pulmonary Fibroblast Proliferation by CAE

**Figure 2:**
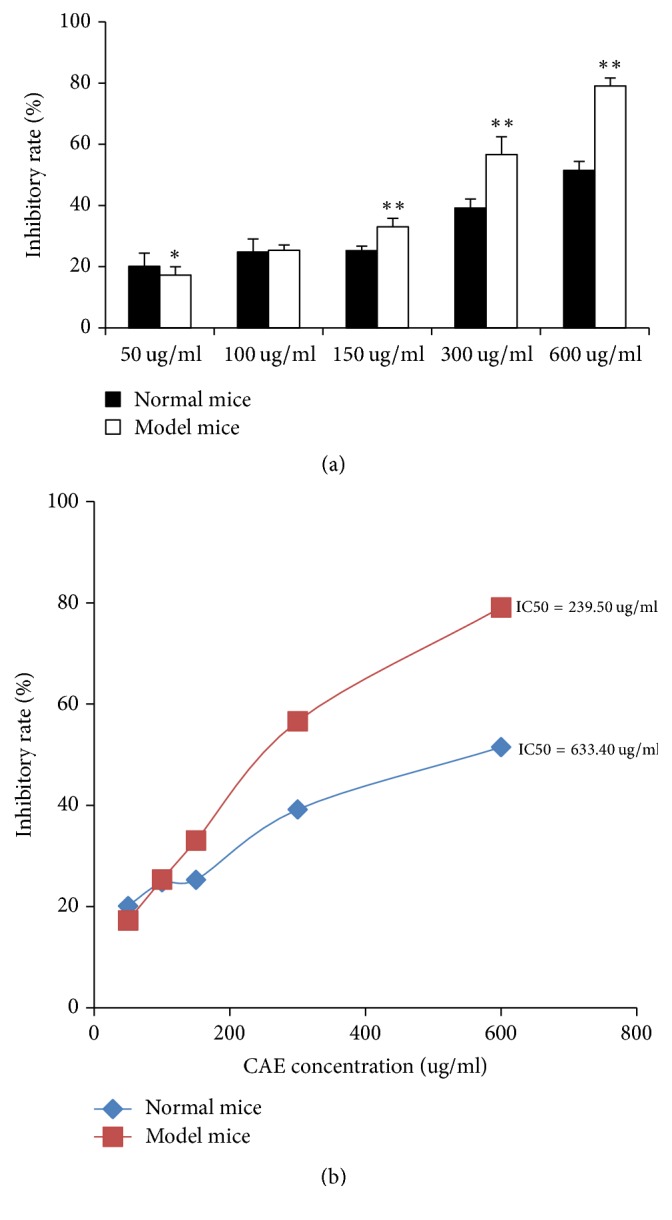
Inhibition of pulmonary fibroblast proliferation by CAE: primary pulmonary fibroblasts treated with CAE at various concentrations for 48 h. The density of pulmonary fibroblasts was adjusted to 1 × 10^5^/ml, and the serum concentration was 5%. CAE was dissolved by DMSO, and the concentration of DMSO in each solution is less than 0.1%. (a) MTT analysis of inhibiting pulmonary fibroblast proliferation by CAE. All data are expressed as mean ± SD (*n* = 6). ^*∗*^*P* < 0.05 versus normal mice, ^*∗∗*^*P* < 0.01 versus normal mice. (b) IC50 value of CAE inhibitory was with respect to the two kinds of pulmonary fibroblasts proliferation. All data are expressed as mean ± SD (*n* = 6).

We added different concentrations of CAE to the two kinds of pulmonary fibroblasts and then evaluated their effects on the proliferation of both pulmonary fibroblasts by MTT test. As shown in Figure 2(a), after 48 h intervention, the proliferation of both kinds of fibroblasts was inhibited efficiently by 50–600 ug/ml of CAE, suggesting a concentration-dependent effect. However, we found that, compared with the pulmonary fibroblasts of the model mice, the inhibition effect of CAE on the pulmonary fibroblasts of the normal mice was enhanced at 50 ug/ml. With the increase of CAE concentration, when CAE dosage was 100 ug/ml or more, its effect on the pulmonary fibroblasts of the model mice was better. We further calculated the half maximal inhibitory concentration (IC50) value after the two kinds of pulmonary fibroblasts were treated by CAE and found that IC50 value of the pulmonary fibroblasts of the normal mice was higher than that of the model mice. It thus suggested an even higher effect of CAE on the pulmonary fibroblasts of the model mice.

### 3.3. LDH Release Test

**Figure 3:**
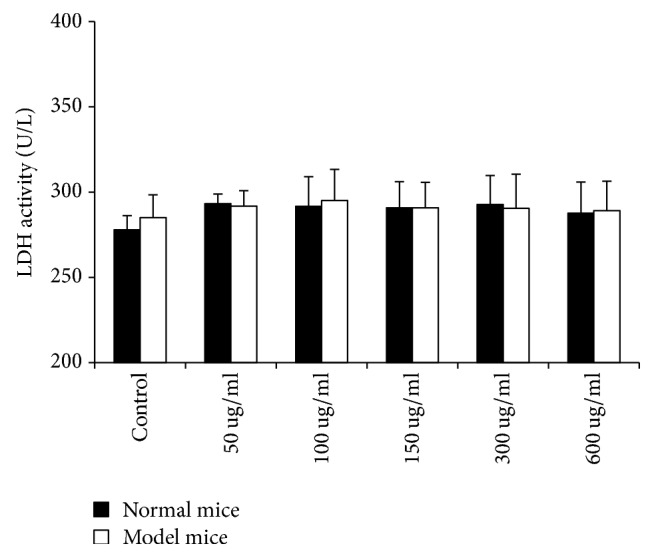
LDH release test: cells were treated with or without varying doses of CAE for 48 h and then the supernatant was collected. All data are expressed as mean ± SD (*n* = 6). No statistical significance was observed between the control group and the CAE-treated groups.

There is ample LDH in cytoplasm, and LDH cannot pass through cell membrane. In case of cell damage or death, LDH leakage can increase LDH activity. Therefore, LDH activity can reflect cell damage or cell death. As shown in Figure 3, the result of the assay revealed that at the concentrations up to 600 ug/mL used in the current study, CAE did not show any cytotoxicity on the two kinds of cells.

### 3.4. CAE Accelerates Pulmonary Fibroblast Apoptosis

**Figure 4:**
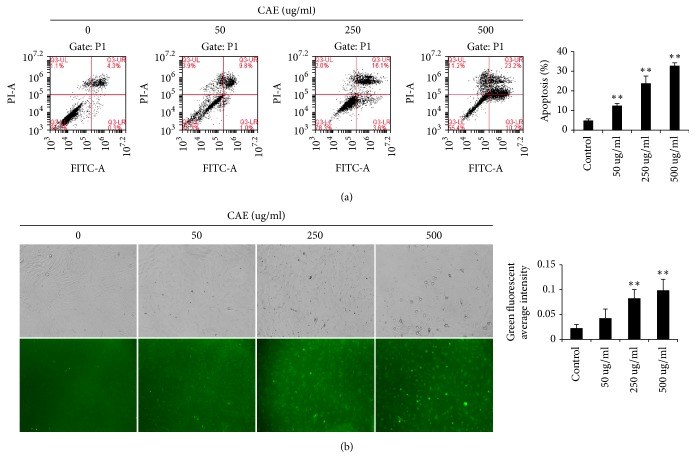
CAE accelerates pulmonary fibroblast apoptosis: (a) flow cytometry analysis. Cells were treated with or without varying concentrations of CAE (0, 50, 250, and 500 ug/mL). After 48 h, the cells were stained by Annexin V-FITC test kit and analyzed by flow cytometry. Histogram indicated that the apoptosis rate in the CAE group was significantly higher than the control group. For the statistics of each panel in this figure, all data are expressed as mean ± SD (*n* = 3). ^*∗∗*^*P* < 0.01 versus control. (b) Cells were stained by Annexin V-FITC test kit and observed with a fluorescence microscope. For the statistics of each panel in this figure, all data are expressed as mean ± SD (*n* = 5). ^*∗∗*^*P* < 0.01 versus control.

Cell apoptosis is characterized by cell wrinkle, chromatin condensation and pyknosis and breakage of nuclei into several round granules with different size [18]. There exists clear apoptosis resistance in pulmonary fibroblast [19]. In this study, in order to determine the effects of CAE on pulmonary fibroblast apoptosis, we tested the apoptosis rate of pulmonary fibroblasts with flow cytometer. The results showed that low (50 ug/ml), moderate (250 ug/ml), and high dosage (500 ug/ml) of CAE all increased pulmonary fibroblast apoptosis to a certain degree and were significantly higher than the control group (Figure 4(a)), suggesting that CAE dosage has markedly promoted pulmonary fibroblast apoptosis. The results of Annexin V-FITC fluorescence staining also showed that with increasing CAE concentration the early apoptosis was profoundly increased (Figure 4(b)).

### 3.5. CAE Promotes the Expression of Apoptosis-Related Signal Protein

**Figure 5:**
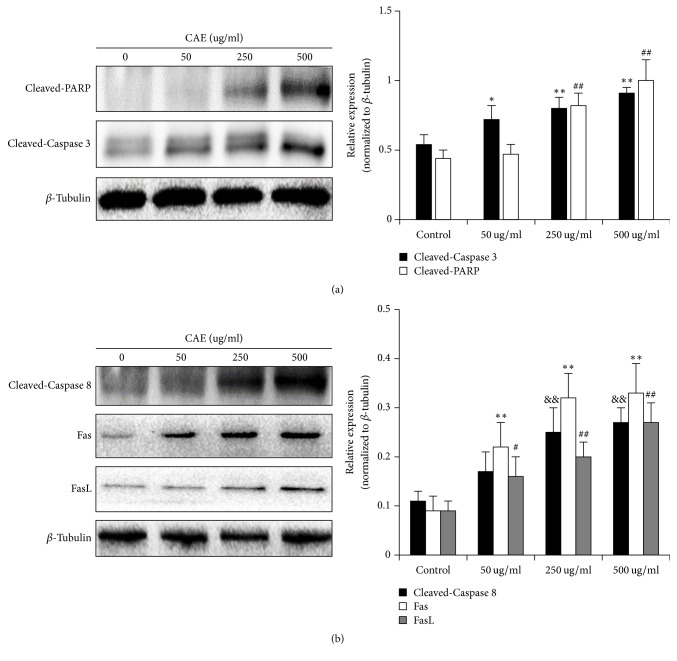
CAE promotes the expression of apoptosis-related signal protein: primary pulmonary fibroblasts treated with or without CAE at various concentrations for 48 h. (a) Western Blot analyses of Cleaved-Caspase 3 and Cleaved-PARP; *β*-tubulin was used as an invariant control for equal loading. Representative blots were from three independent experiments. Data are expressed as mean ± SD (*n* = 3). ^##^*P* < 0.01 versus control; ^*∗*^*P* < 0.05 versus control; ^*∗∗*^*P* < 0.01 versus control. (b) Western Blot data showing the protein expressions level of different apoptosis-related protein (Cleaved-Caspase 8, Fas, and FasL). Data are expressed as mean ± SD (*n* = 3). ^#^*P* < 0.05 versus control, ^##^*P* < 0.01 versus control; ^*∗∗*^*P* < 0.01 versus control; ^&&^*P* < 0.01 versus control.

We examined how pulmonary fibroblast apoptosis was promoted by CAE in this paragraph. Caspase 3 is the performer protein of cell apoptosis. We assayed the expression level of Cleaved-Caspase 3 using Western Blot. As shown in Figure 5(a), CAE significantly activated Cleaved-Caspase 3 in a concentration-dependent way. The effect of CAE was more significant at its moderate and high concentrations. PARP is the substrate of Caspase 3, so its activity can reflect the degree of cell apoptosis to a certain degree. When moderate or high dosage of CAE was added, the activation degree of Cleaved-PARP was more significant.

The pathway of cell apoptosis includes membrane receptor apoptosis, mitochondria apoptosis, and endoplasmic reticulum apoptosis. But we do not know in which pathway CAE has caused pulmonary fibroblast apoptosis. For this purpose, we studied the effects of CAE on the expression of Cleaved-Caspase 8, Cleaved-Caspase 9, and Cleaved-Caspase 12. We found that CAE only promoted the expression of Cleaved-Caspase 8 but did not have obvious effect on Cleaved-Caspase 9 and Cleaved-Caspase 12 (Figure 5(b)). This suggested that CAE probably has affected pulmonary fibroblast apoptosis through the membrane receptor pathway. Caspase 8 is the sponsor of membrane receptor apoptosis and is regulated by its upstream Fas and its ligand FasL. We therefore analyzed the expression of Fas and FasL and found that CAE promoted the expression of Fas and FasL in a concentration-dependent manner (Figure 5(b)). The results above suggested that CAE-induced pulmonary fibroblast apoptosis is realized by Fas mediated membrane receptor apoptosis.

### 3.6. Activation of CAE-Induced Membrane Receptor Apoptosis Pathway Is Related to the Regulation of COX-2

**Figure 6:**
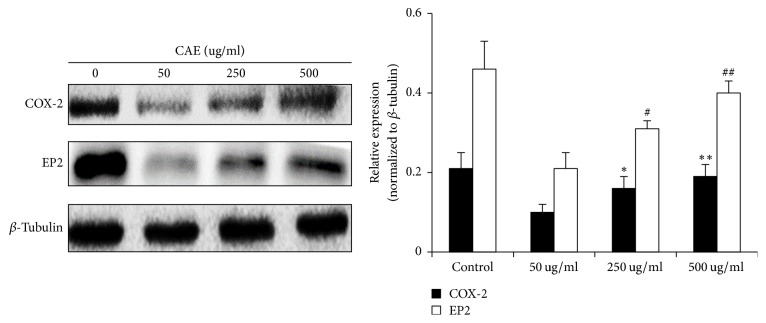
Activation of CAE-induced membrane receptor apoptosis pathway is related to the regulation of COX-2: the expression of COX-2 and EP2 was dependent on CAE concentrations, but all were lower than the control group. Data are expressed as mean ± SD (*n* = 3); ^#^*P* < 0.05 versus CAE-50 ug/ml group, ^##^*P* < 0.01 versus CAE-50 ug/ml group; ^*∗*^*P* < 0.05 versus CAE-50 ug/ml group, ^*∗∗*^*P* < 0.01 versus CAE-50 ug/ml group.

How can CAE induce the activation of membrane receptor apoptosis pathway? What is the functioning mechanism? It is believed at present that COX-2 is a specific catalyzing enzyme that plays a protecting role in the occurrence and development of pulmonary fibrosis. It can activate PGE2 and further activate Fas, thereby initiating the membrane receptor apoptosis pathway, by acting on membrane receptor, mainly EP2. Therefore, we further asked whether CAE-activated membrane receptor apoptosis was related to the COX-2 signaling pathway. Results of Western Blot showed that the expression of COX-2 and EP2 was dependent on CAE concentrations, but all were lower than the control group (Figure 6). However, we could not detect distinct expression of PGE2 due to tiny amount of expression. Therefore, the occurrence of CAE-induced membrane receptor apoptosis pathway is probably related to the activation of COX-2-related signaling pathway.

### 3.7. CAE Accelerates COX-2 Expression through Oxidative Stress Response and MAPK Signaling Pathway

**Figure 7:**
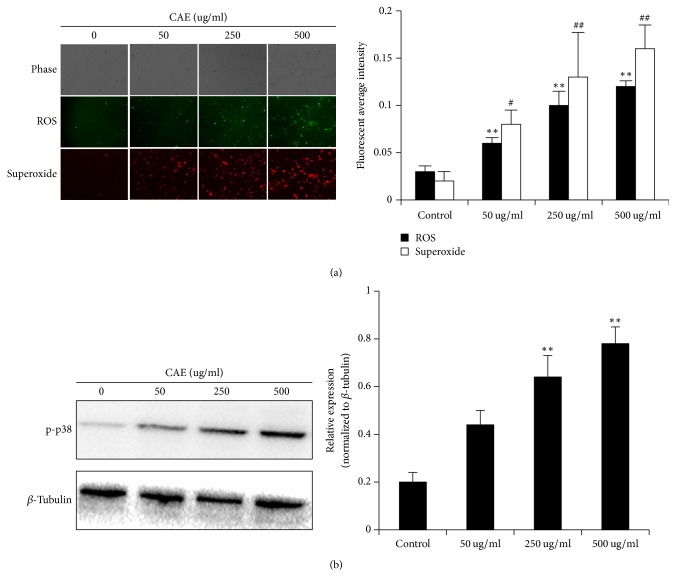
CAE accelerates COX-2 expression through oxidative stress response and MAPK signaling pathway: (a) cells were stained by oxidative stress test kit and observed with a fluorescence microscope. For the statistics of each panel in this figure, all data are expressed as mean ± SD (*n* = 5). ^*∗∗*^*P* < 0.01 versus control; ^#^*P* < 0.05 versus control, ^##^*P* < 0.01 versus control; (b) Western Blot analyses of p-p38; *β*-tubulin was used as an invariant control for equal loading. Data are expressed as mean ± SD (*n* = 3); ^*∗∗*^*P* < 0.01 versus control.

In which pathway COX-2 protein is activated by CAE? It has been shown that the activation of COX-2 is probably directly related to oxidative stress. When the oxidative stress response is activated, protein phosphorylation relevant to the MAPK signaling pathway is increased, thereby accelerating the expression of COX-2. To test this assumption, we measured the oxidation response using ROS test kit. The results showed that CAE increased oxidative stress response in a concentration-dependent manner (Figure 7(a)), suggesting that CAE can activate the oxidative stress response. We then measured the phosphorylation level of ERK (p-ERK), JUK (p-JUK), and p38, three phosphorylated proteins in the MAPK signaling pathway. As shown in Figure 7(b), CAE increased significantly the expression level of p-p38, but that of p-ERK and p-JUK remained unchanged. This suggested that CAE regulated the activation of COX-2, which was related to the oxidative stress response and the expression of p-p38.

## 4. Discussion

Pulmonary fibrosis is a progressive lethal disease. Its prevalence keeps increasing year by year, and no medicine has been proven effective in pulmonary fibrosis treatment. Therefore, it is of important clinical value to seek for medicine with low side effects and high safety and efficiency in pulmonary fibrosis treatment. Our previous study found that citrus alkaline extract could effectively prevent bleomycin-induced pulmonary fibrosis in rat and inhibit pulmonary fibroblast of human embryo [20]. In this paper, we constructed a pulmonary fibrosis mouse model using bleomycin induction and then tested the therapeutic effect of CAE using the primary mouse pulmonary fibroblasts and further confirmed its functioning mechanism.

At present, pulmonary fibrosis model can be constructed by several methods, including paraquat and amiodarone [21, 22]. But the frequently used method is intratracheal instillation of bleomycin [23]. A central step of pulmonary fibrosis process is pulmonary fibroblast. The primary pulmonary fibroblast is isolated from pulmonary cells and cultivated* in vitro*. It can better reflect the growth trait of pulmonary fibroblasts. In this study, we extracted the pulmonary fibroblast of the normal and model mice and then determined the expression of vimentin and *α*-SMA through the immunohistochemical method. The results showed that the positive expression rate of vimentin in both fibroblasts did not differ significantly, but the expression intensity of pulmonary fibroblasts in the model mice was higher than that in the normal mice. Meanwhile, the positive expression rate and expression intensity of *α*-SMA of the pulmonary fibroblasts in the model mice were also higher than those in the normal mice, which suggests a successful model construction. Furthermore, the bleomycin-induced mouse pulmonary fibroblast was much closer to human pulmonary fibroblast, which facilitates studies.

To verify the effect of CAE on pulmonary fibroblasts, we firstly tested cell viability using MTT method. The quantity and viability of pulmonary fibroblasts can be reflected indirectly by the absorption value at 540 nm. We found that different concentrations of CAE had markedly inhibited the two kinds of pulmonary fibroblast activity and changed their morphology. We further calculated the IC50 value after the two kinds of fibroblasts were treated by CAE and found that IC50 value of the pulmonary fibroblasts of the normal mice was higher than that of the model mice. It thus suggested an even higher effect of CAE on the pulmonary fibroblasts of the model mice.

Meanwhile, to exclude the direct killing power of cytotoxicity to pulmonary fibroblasts, we used LDH to test the cytotoxicity. Results showed that the inhibition effect of CAE on the two kinds of pulmonary fibroblasts was not related to cytoxicity, suggesting an ideal inhibition of CAE on the activity of pulmonary fibroblasts.

The results confirmed the higher value of the pulmonary fibroblasts of mouse model and there was an even higher effect of CAE on the pulmonary fibroblasts of the model mice, so we only used the pulmonary fibroblasts of mouse model in the further research. In order to specify the mechanism by which CAE inhibits pulmonary fibroblasts, we observed morphological change of pulmonary fibroblasts and even death with microscope after CAE treatment. There are several ways for cell death. We however focused on cell apoptosis and tested whether if CAE can induce pulmonary fibroblast apoptosis. Quite a few methods can be used to test cell apoptosis. We found that cell apoptosis was increased after different concentrations of CAE were added based on flow cytometer analysis. The results of Annexin V-FITC fluorescence staining also supported this conclusion. The results of WB showed that expression level of two characteristic proteins in apoptosis, Cleaved-Caspase 3 and Cleaved-PARP, was significantly increased after CAE addition. Therefore, CAE did induce pulmonary fibroblast apoptosis.

It is now known that there are three pathways that regulate cell apoptosis. The first is the death receptor-regulated membrane receptor apoptosis pathway. In this pathway, Caspase 8 is activated by Fas and its ligand FasL, and then it activates the membrane receptor apoptosis pathway [24]. The second is mitochondria apoptosis pathway. In this pathway, the apoptosis protein Bax interacts with the antiapoptosis protein Bcl-2. Both proteins regulate Caspase 9 and activate the mitochondria apoptosis pathway [25]. The third is the endoplasmic reticulum apoptosis pathway wherein C/EBP homologous protein (CHOP) and other related proteins participate into the activation of Caspase 12 [26]. Current research showed that Caspase 8, Caspase 9 and Caspase 12 are the promoters of these three apoptosis pathways. In order to confirm CAE-induced pulmonary fibroblast apoptosis belongs to which apoptosis pathway, we assayed the expression of three proteins by Western Blot. Our results showed that CAE had significantly increased the expression of Cleaved-Caspase 8, while the expression of Cleaved-Caspase 9 and Cleaved-Caspase 12 has not been affected. This suggested that CAE-induced pulmonary fibroblast apoptosis was probably involved with the membrane receptor apoptosis pathway. To further confirm this conclusion, we determined the expression of Fas and FasL, two key membrane proteins that regulate membrane receptor apoptosis. The results of WB also showed that CAE has significantly increased the expression of Fas and FasL. Therefore, CAE-induced pulmonary fibroblasts are probably involved with the membrane receptor apoptosis pathway.

In pulmonary fibrosis, the membrane receptor apoptosis is regulated by a number of factors. It has been reported that COX-2 can regulate the membrane receptor apoptosis pathway [27]. COX-2 is a PGE2-specific upstream kinase that promotes PGE2 production. PGE2 binds to its four receptors (EP1, EP2, EP3, and EP4), enters cell, and functions therein. Of these four receptors, EP2 plays the most important roles [28]. COX-2 is traditionally believed to promote the inflammatory response in pulmonary fibrosis. In recent years, it was found that the function of COX-2 in pulmonary fibrosis is not proinflammatory but anti-inflammatory. Its role in pulmonary fibrosis is protective which inhibits the development of pulmonary fibrosis [28]. Studies have proven that COX-2 can increase the expression of pulmonary fibroblast Fas in pulmonary fibrosis patients and accelerate pulmonary fibroblast apoptosis. Other studies found that COX-2-mediated Fas/FasL signaling pathway not only accelerates pulmonary fibroblast apoptosis, but also inhibits the apoptosis of pulmonary epithelial cells [27]. We therefore speculated that Fas/FasL-mediated pulmonary fibroblast apoptosis is probably closely related to COX-2. To test this assumption, we tested the expression of corresponding proteins by Western Blot. Results showed that the COX-2 and EP2 in pulmonary fibroblasts were highly expressed. When pulmonary fibroblasts were exposed to different concentrations of CAE, the expression of COX-2 and EP2 showed a concentration-dependent increase, although their expression was lower than the control group. In the study, the expression level of pulmonary fibroblast COX-2 and EP2 in the treatment group was very high. This is inconsistent with the results of the primary cells in idiopathic pulmonary fibrosis patients [28]. A possible reason is that although bleomycin-induced pulmonary fibrosis mice were used to study pulmonary fibrosis, it is more close to an acute pulmonary wounding model. The inflammatory response appeared throughout the study. Therefore 28 days after model construction, the inflammatory response was still present. After CAE intervention, the expression of COX-2 increased with increasing CAE concentration. This suggested that CAE intervention has controlled the inflammatory response on one hand and that it also activated the protecting role of COX-2 in pulmonary fibrosis on the other hand.

A wealth of studies showed that the regulation of COX-2 in pulmonary fibrosis is affected by many factors. Study results in recent years showed that oxidative stress response can regulate COX-2 protein through the activation of related signaling pathway, for example, MAPK signaling pathway [29, 30]. The MAPK family of mammals includes mainly three categories, p38, JNK, and ERK. Different signaling pathway in the MAPK family can be activated by different stimulus, leading to corresponding biological effects [31]. It was reported that when human embryo pulmonary fibroblast (IMR-90) was exposed to H_2_O_2_ to induce oxidative stress injury, the expression of P38 and COX-2 was significantly increased. But when P38-specific inhibitor SB203580 was added, the oxidative stress response was weakened and the expression of COX-2 was markedly reduced [30]. This suggested that p38 has functioned to regulate COX-2 in oxidative stress response. It was also found that when pulmonary fibroblast apoptosis was induced by drugs, the initially activated response was probably the Redox reaction, and a center that controls drug-induced pulmonary fibroblast apoptosis is the MAPK-COX-2-related signaling pathway [10]. To verify whether CAE could induce oxidative stress response in pulmonary fibroblasts, we tested it using the ROS test kit. The results showed that when ROS and superoxide were added the fluorescent reaction was enhanced significantly, suggesting an increased oxidative stress response. We then verified whether oxidative stress response was related to the MAPK signaling pathway. Results showed that the expression level of p-p38 was markedly increased, suggesting that p38 probably have participated in CAE-induced oxidative stress reaction.

## 5. Conclusions

To sum up, we constructed a BLM-induced pulmonary fibrosis mouse model and extracted the primary mouse pulmonary fibroblasts. We found that CAE can induce pulmonary fibroblast apoptosis. A possible functioning mechanism is that CAE activates the oxidative stress response, which may promote the p-p38, activate COX-2, accelerate Fas expression, initiate the membrane receptor apoptosis pathway, and eventually induce pulmonary fibroblast apoptosis. The study is the basic study in pulmonary fibrosis, which may supply some theoretical evidence to clinical therapy. However, the specific mechanism of CAE still needs more in-depth investigation.
